# Annual Meeting

**Published:** 1888-02

**Authors:** 


### ILLINOIS STATE BOARD OF HEALTH.

ANNUAL MEETING, SPRINGFIELD, JANUARY 12–13, 1888.

The Eleventh Annual Meeting of the Illinois State Board of Health was held in Springfield on January 12 and 13, 1888. The Secretary presented the Quarterly Report and Annual Summary. In it he said, that in view of the very general interest which had been aroused throughout the country by the recent occurrences at the New York quarantine and the recent apparent danger of an immediate invasion of Asiatic cholera through Italian immigrants, it seemed proper to give that subject precedence in the Quarterly Report. A history of the introduction of the contagion into New York Harbor in the fall of 1887, and of the action of this Board in the premises, was given.

From this it seemed evident that the Board shares the general belief that sufficient care was not taken at the quarantine of New York regarding infected vessels (the Alesia and the Britannia), which arrived at that port during the autumn of 1887.

In this report the Secretary says, regarding public health and the immigrant: These occurrences and the correspondence thereon very opportunely illustrate and emphasize the position I have held and the views I have promulgated upon the subject of quarantine for many years. Illinois, through the authority vested in its State Board of Health, may be protected against the consequences of the lapses and defects of the coast quarantines; but at a wholly unjustifiable expense and a regrettable but necessary interference with transportation interests. No interior State should be compelled, in order to protect the health and lives of its citizens against foreign pestilence, imported through the seaboard cities, to maintain a State line quarantine, to inspect the passengers on railways from such cities, and otherwise to do, at great disadvantage and under very unfavorable conditions, what might much more readily, cheaply, and efficiently be done at the port of arrival. The Illinois State Board of Health has exercised its quarantine power only as a last recourse; and, when this exercise has been unavoidable, every effort has been made to adjust its inspections and other agencies so as to conflict as little as possible with the interests of commerce and travel. On the other hand, the railroad managements have always responded to the Board in the most amicable spirit, with an intelligent appreciation of the necessities of the situation and a ready extension of every facility in their power in order to accomplish the desired result with as little friction as possible.

But the question naturally arises: Why should the State of Illinois be put to this expense, and with what justice can her transportation agencies be subjected to this burden? It is not her own protection alone that is thus assured, but the protection of the vast region beyond her borders—northwest, west, and southwest—to which the great tide of immigration flows. Of the thousands of immigrants who enter the State annually—mainly through Chicago as the great distributing point—only a small portion remain as permanent settlers; but the diseases, contagious and infectious, as well as the moral, social, and mental wrecks of the whole number, are inflicted upon us in vast disproportion.

This consideration, however, is swallowed up in the larger view of the interests of the whole country, and the duty of the National Government to protect its borders from the invasion of foreign pestilence. And with regard to Asiatic cholera—whatever may be true of small-pox, scarlet fever, or other contagious diseases—the events of the past few months only corroborate my conclusions; they have not modified, reversed, or added thereto. I repeat what I have so often said before, as a practical sanitarian accustomed to deal with conditions as they actually exist, to wit: That the only wise thing to do in respect of cholera is to resist the first beginnings. This country can be protected against the disease only by its absolute exclusion. The arguments against quarantine which may be valid enough for Great Britain do not apply here. The conditions which obtain are widely different. There is no great immigration movement into that country such as complicates the social, political, and sanitary problems of this country. The only large bodies of people arriving in England after a long voyage are those on the troopships; but these are constantly under medical and sanitary surveillance, and the necessary measures may be enforced under the direct control of government.

The vast hordes of immigrants constantly arriving in this country present a sharp contrast in every respect. The voyage across the Atlantic is long enough to increase the susceptibility to contagious diseases under the conditions which obtain in the steerage; the sanitary supervision is defective; reports of the ship's officers are untrustworthy, as is again shown in the case of the Britannia; and the methods of the immigrant carriers—whose object is to land the immigrant at his ultimate destination with as little delay as possible—are such as to greatly imperil the interior.

The immigrant and his effects are hurried through the port of arrival, and the first intimation of danger to an interior community is too often the outbreak of a virulent contagion. I have shown this to be true with respect to small-pox, in my paper entitled "Immigrant Introduction of Small-Pox into the United States." (See Fifth Annual Report of the Illinois State Board of Health, pp 329–342. Also report of the National Board of Health, 1882.) During a period of nearly a third of a century I have traced the reappearance of small-pox in Chicago in every instance to first cases directly introduced by immigrants; and the first great small-pox epidemic—that of 1880–83—I have shown to be due to the same cause in ten different States and in over two hundred different instances.

The familiar citation of the epidemic outbreaks of cholera in 1873 in Ohio, Minnesota and Dakota—caused by the unpacking of the infected household goods, clothing, etc., of immigrants—might be supplemented by similar instances which came under my own observation during that year, while Sanitary Superintendent of Chicago. The first cases in that city were in three different families, who had received immigrants from Europe direct through the port of New York. (There was no cholera in New York City in 1873.) Members of these families were attacked by the disease within a short time after the arrival of these persons. The fact that the immigrants themselves escaped, threw the search for the origin of the cases into other directions; and I vainly sought to establish a connection between these outbreaks and the places then infected on the Mississippi River. It was not until after such connection was positively disproved that the right clue was discovered as above mentioned.

In view of the subsequent developments in the case of the Alesia and Britannia it can only be ascribed to great good fortune that the country escaped a similar result from her immigrant passengers. The number of cases which occurred after these were taken in charge by the quarantine authorities and the prolonged duration of the outbreak are conclusive proof that they were infected, not only in person but in belongings. If there had not been cases of the disease actually on board when the vessel arrived these infected belongings—baggage, dunnage, household effects, etc., packed up in the cholera districts of Italy

—would have been passed through the port and rapidly carried into the interior, there to liberate the cholera poison whenever the goods were opened, as was the case in 1873 at Carthage, Ohio, in Kandiyohi County, Minnesota, at Yankton, Dakota, and in the city of Chicago.

While this demonstrates the necessity for the thorough disinfection of all this class of articles coming from any country infected with such a transportable contagion, the cases of cholera which continued to occur among the steerage passengers of the Alesia and Britannia so long after arrival equally demonstrate the necessity for detaining such passengers during the recognized period of incubation of the disease. Given the existence of cholera in Europe, experience shows the impossibility of determining when or how the immigrant passengers may become exposed to infection during a voyage.

The terrible epidemic of 1848–50, which swept over the continent from the Atlantic to the Pacific, is attributed to the arrival at New Orleans in December, 1848, of the emigrant ship Swanton. This vessel cleared from the port of Havre on the 31st of October; and nine days later the New York—another emigrant ship—cleared from the same port for the port of New York. Havre was at the time declared free from cholera, and both vessels cleared with clean bills of health. According to Hirsch cholera did not invade France until the first months of the following year, when it first showed itself in the seaports along the northern coast. The steerage passengers on these vessels, however, were mostly emigrants from Germany, into which country—according to the same authority—cholera was brought from Russia in the early summer of 1848.

On the 24th of November, being then fifteen days out from Havre with all well on board, the New York, in north latitude 42° off the United States coast, encountered a very cold wind which, in the language of the captain, caused "a general overhauling of baggage for warm clothing." Twenty-four hours later the Swanton, a thousand miles south of the New York (in latitude 25° 47′) was "visited by a very hot south-east wind; such a one," says her captain, "as I had never felt before." Cholera broke out on the New York November 25, and on the Swanton November 26. The former vessel arrived in New York Bay December 2, having lost fourteen of her passengers, and the latter reached New Orleans December 11, having had thirteen deaths.

There is no escape from the conclusion arrived at by Dr. Alonzo Clark, after a critical consideration of the various theories advanced to account for the outbreaks on these vessels: "It seems to me far more probable that the poison was lurking in the baggage of these passengers, and that the emergencies which required the opening of unventilated trunks and packages, let loose in each of these ships that poison brought from the infected regions of Germany."

Had this unpacking of baggage occurred later in the voyage the vessels might have arrived without any suspicion of infection; and under the present system of local maritime quarantines, they would at most have been detained only long enough to disinfect the vessel, baggage, etc. Manifestly, since it is impossible to disinfect the individual, who may already have received the seeds of contagion into his system, the only security for the country is in the detention —*for the full period of incubation* after arrival—of all European immigrants so long as Asiatic cholera continues to exist in any part of Europe.

### PROHIBITION OF IMMIGRATION.

The alternative to these precautions is in the prohibition of all immigration from cholera infected districts during the existence of a cholera epidemic. This might be done by giving the President authority to issue a proclamation to such effect, as I have already suggested in my address before the National Conference of State Boards of Health in 1884.

The occurrences of the past few months fully confirm all that I have hitherto said upon this subject. Maritime quarantine can be efficiently administered only by the National Government, and its cost should be defrayed from the national treasury, since it is a measure of sanitary defense for the whole country against the invasion of foreign pestilence. A short cut to the same result may be had by shutting out the immigrant himself, and with him all the other ills which the steerage, like a veritable Pandora's box, has let loose upon this country. As I have elsewhere said: It is the steerage of the immigrant vessel, with the poverty, squalor, and uncleanliness of too many of its denizens, with its crowd-poison and other conditions favorable to the development of the specific contagions, which we, as sanitarians at least, have to fear and guard against.

If Congress can be induced to give the subject the consideration which its importance to the well-being of the Republic demands, it would be well to inquire into the feasibility of restricting the entrance of immigrants to those ports only which may be provided with proper facilities for their inspection, detention when necessary, and other treatment adequate to effectually prevent their carrying contagion into the country either by themselves or their belongings. And in this connection it seems proper to observe, in view of recent developments, that the language employed in my "Report of an Inspection of the Atlantic and Gulf Quarantines," with reference to the quarantines at New York and at New Orleans, was well considered. I then, in the fall of 1885, wrote: The quarantine plant and facilities at the port of New York are unrivaled, etc. That the "plant" has not been maintained in the condition it was when first constructed, and that the "facilities" have not been utilized, do not surprise those familiar with the situation. The result witnessed in the fall of 1887 is foreshadowed in the concluding paragraph of my report on this station: "New York *may* exclude cholera under her present system; but more confidence would be reposed in the result if less were demanded of professional ability, personal integrity, and executive firmness in the health officer, and if the system were freed from influences which are most deprecated by those who, without prejudice, best understand them." In short, the sanitary efficiency of the New York quarantine has been interfered with to a serious extent by political influences.

With regard to New Orleans, I still indorse the claim made that it "is the most thorough and vigorous system of sanitary quarantine which has ever been enforced for the protection of a port from the introduction of foreign contagion." But from what has been already said in the preceding it is obvious that I do not believe that any amount of sanitation—of disinfection, cleansing, etc.—will suffice to exclude such a capricious disease as cholera. There is absolutely no safety for this country in allowing immigrants from Europe—so long as cholera exists anywhere on that continent—to land on our shores until having complied with the following conditions:

First. The vessel to be taken charge of by the quarantine authorities immediately on arrival, and released only after she is put in a perfect sanitary condition.

Second. The thorough cleansing and disinfection of all personal belongings of passengers, officers, and crew—*coupled with a detention of observation long enough to cover the recognized period of incubation of the disease after arrival.*

Fortunately there have been, as yet, no cases of cholera developed among or from the immigrants allowed to land and travel into the interior from these various vessels, and it is probable that whatever danger there may have been from these is now past. Occasional cases of cholera still occur in southern Europe, and experience, both remote and recent, forbids a positive conclusion that the epidemic has run its course in that country; while we know that it has again reappeared in Chili, and is now raging at Santiago, and its extent in South America can only be conjectured.

WATER SUPPLIES AND THE POLLUTION OF STREAMS.

Turning from this consideration of the subject to our own duty as a State Board of Health, the occurrences above dwelt upon fully justify all the efforts made during the past four years to perfect the sanitary condition of the State, and emphasize the necessity for their further continuance. The results of the unparalleled drought of the past year demonstrate the importance of a vigorous prosecution of the investigation into the sources of and remedy for the pollution of streams and the character of the water supply, not only of cities, towns, and villages, but of the country generally throughout the State. Coupled with the chemical and biological examination of water supplies, it is designed to utilize the data obtainable by collecting the various borings made in many parts of the State for coal, artesian wells, gas, etc. These should indicate the character, extent, and availability of our sub-surface water supply, and the results of the study and research, while being directly in the line of sanitary interests, will be profitable from an economic standpoint. Only less important than the prevention of disease, and premature death thereby, would be the knowledge which should enable the farmers, stock-raisers, and others to remedy, as far as possible, the results of prolonged dry weather, and to supplement the ordinary sources of supply from the underground reservoirs.

Illinois, though suffering, has, fortunately, been spared the widespread diseases which have been caused by drought during the past year in neighboring States, but the mode of cholera propagation and the extent and severity of a cholera epidemic depending so largely upon the character of the water supply, we cannot rest satisfied until every community in the State is assured of the best attainable condition of this great sanitary necessity. Wherever typhoid fever spreads beyond sporadic cases, and wherever diarrhœa and dysentery are usually prevalent, it is safe to assume that Asiatic cholera may become epidemic if the specific contagion be introduced, and it will be wise to remedy the conditions which cause such spread and prevalence before the graver disease shall get a foothold.

SANITARY SURVEYS.

In addition to prosecuting this work during the coming season, the general sanitary labors, which have been on the whole satisfactorily pursued during the past year, will need to be stimulated and encouraged during 1888. Sanitary surveys of places, where these have not yet been undertaken, and revision and utilization of those already made, are among the most useful agencies which the Board has yet devised for promoting general sanitation.

During the past year the surveys of Alton, Anna, Belleville, Beardstown, Carlinville, Collinsville, Freeburg, Mt. Vernon, Ottawa, Rockford, Rock Island, Springfield, Streator, Tuscola, and Urbana have been completed, and are now in possession of the Board awaiting publication. The data contained in these surveys are not confined to sanitary matters alone, but there is much information pertaining to every locality which has never before been collected, and is nowhere else accessible. There is a marked improvement in the completeness and thoroughness of the work done during the past year in this direction. This is, no doubt, largely due to the publication of the sanitary surveys in the eighth annual report of the Board. These have obviously been studied and profited by, and it is a source of regret that the publication of the ninth annual report —the matter for which has been for some time ready for the printer –has been so long delayed through the complications in the public printing.

HEALTH OF THE STATE.

Localized outbreaks of diphtheria and scarlet fever have been reported from a number of places during the closing quarter of 1887, but with no special tendency to epidemic spread. Want of proper and efficient municipal sanitary organization has prolonged the existence of the former disease in Belleville and of the latter in Quincy. Diphtheria is reported at Beardstown, Belleville, Blair, Edwardsville, Elmhurst, Nokomis, Rockford, Sadorus, and near Sparta; scarlet fever at Chester, Mount Sterling, Nokomis, Quincy, Rockford, Sannemin, and Sparta; typhoid fever and "milk sickness" near Nokomis and neighborhood of Potomac, and a group of cases of typho-malarial fever at Downs.

A case of small-pox developed in the person of a railroad conductor on his return to Clinton (DeWitt County), from an excursion into Mexico, where he undoubtedly contracted the disease. A case of varioloid has also occurred at Decatur. The source of the contagion in this latter case has not yet been determined. The usual precautions prescribed in the preventable-disease circular of the Board have been enforced at these places, and the local authorities do not anticipate any spread of the contagion at Decatur. The conductor at Clinton is convalescent, but his three children have contracted the disease. Every effort is being made to confine the disease to this one family.

Another edition of the circular on scarlet fever, with some few revisions, has been prepared during the quarter and is now ready for distribution.

MEDICAL-PRACTICE ACT.

There were issued during the quarter ninety-four certificates authorizing the practice of medicine in the State—sixty-six to graduates upon diplomas from medical colleges in good standing; twenty-four to non-graduates upon proof of ten or more years' practice in the State prior to July 1, 1887, and four duplicates upon proof of the destruction of the originals. This number is larger than usual in the last quarter of the year, the increase being due to the operation of the revised act by which those formerly exempt are now required to take out certificates.

Certificates were also issued to twenty midwives—ten upon diplomas or licenses; two upon examination; seven upon years of practice, and one duplicate.

During the year four hundred and eighty-five original and thirteen duplicate certificates authorizing the practice of medicine were issued. Diplomas presented by thirty-one graduates of seven different colleges were required to be supplemented by examinations of the applicants on branches or subjects omitted by the respective schools; and in six similar cases the applicants declined to appear for examination and left the State. In addition to these, sixty-nine applications were refused owing to the inability of the applicants to comply with the terms of the medical-practice act, or the rules of the Board based thereon.

Of the total four hundred and eighty-five original certificates issued, four hundred and thirty-four were to graduates of medical colleges; forty-four to practitioners of ten or more years' practice prior to July 1, 1877, and seven to candidates upon examination. The certificate of one practitioner was revoked for "unprofessional and dishonorable" conduct.

To midwives during the year ninety-five original certificates were issued—thirty-six being to licentiates; thirty-six upon examination, and twenty-three upon years of practice in this State. Seventeen applications were refused, mainly because of failure to pass the prescribed examination.

MEDICAL EDUCATION.

In order to remedy, as far as possible, the effects of the delay in the public printing—the cause of which has been already referred to—the following summary and tables are submitted from the report on medical education, which has formed a feature in the annual reports of the Board for several years past. The matter for this report, which has involved a great amount of labor, is ready for the printer, and as the value of its information would be materially impaired by further delay it is deemed proper to make it public, to this extent at least, in the abstract here presented.

In brief, the data collected indicate a continued improvement in the methods and a still further heightening of the standard of medical education in this country—a marked and steady impetus to which, it may be fairly claimed, has been given by the Illinois State Board of Health in its practical enforcement of a schedule of minimum requirements for medical colleges, conformity with which is necessary to ensure recognition as in "good standing" for the purposes of the medical-practice act; also the annual publication of this report, the work of other boards, and the agitation of the profession in the same direction.

The text of the Report on Medical Education for 1887 contains the data of historical interest pertaining to 258 institutions, dating back to 1765; the last report contained such data for 253 institutions. It contains, also, all necessary information as to the title, location, addresses of corresponding officers, faculty, curriculum of study, length and dates of sessions, requirements for matriculation and for graduation, fees, number of matriculates and of graduates at each session since 1877—and other facts of interest concerning the 129 medical colleges now in existence. It also embraces the statistics of a total of 116,529 medical students, classified by schools of practice, by States, by individual colleges, and by sessions, for each year during the period 1877-78 to 1886-87 inclusive.

The changes noted by a comparison with the report of 1886 show a net gain of two regular schools and a loss of two eclectic schools; while the others remain unchanged. As compared with the conditions which existed prior to the enforcement of the schedule of minimum requirements—that is, prior to the session of 1883-84—the report shows that there are now 114 colleges which exact an educational requirement of intending matriculates, as against 45 formerly—there being no change in this respect from the previous year; that 43 colleges now exact attendance upon three or more courses of lectures as against 22 formerly—being a gain of two over 1886—and 57 others make provision for a three or four years' graded course; that hygiene is now taught in 114, and medical jurisprudence in 112 colleges, as against 42 and 61 respectively, prior to 1883. There is an increase in the average of lecture-terms from 23.5 weeks to 24.9 weeks during this period, and 114 colleges now have terms of five months or over, and 63 have terms of six months or over as compared with 101 and 42 respectively. There is only one medical college that has a course less than twenty weeks—the Medical College of Georgia.

The effect of this improvement in methods of instruction and of the higher qualifications demanded before graduating the student is best seen in the diminishing percentage of graduates to matriculates. The tables embraced in the report now cover a sufficiently long period—from 1877 to 1887 inclusive—and deal with large enough numbers—116,529 matriculates and 37,493 graduates—to give them a positive value for this purpose. Errors arising from unusual or accidental conditions are largely canceled or diminished in importance in these aggregates, and the inferences drawn may be accepted as fairly trustworthy.

The first fact of importance which arrests the attention in this comparison is that, notwithstanding the growth of population, the total number of medical students has never yet reached the proportions attained in 1882-83. The sessions of that winter were attended by 13,088 students; of 1883-84 by 12,763 students; of 1884-85 by 11,975 students; of 1885-86 by 12,321 students, and of 1886-87 by 12,948 students.

Although there was a gain last year of 627 students over the attendance in 1885-86, the aggregate in 1886-87 was still 140 less than in 1882-83.

The effect of a higher standard of qualifications for graduation is further shown in the diminishing percentage of graduates to the total number of students. In 1882-83 out of every 1,000 matriculates 322 were graduated, taking both the United States and Canada and all schools of practice into the account. In 1886-87 only 294 out of every 1,000 matriculates were graduated. In the United States alone in 1882-83 out of every 1,000 matriculates 331 were graduated, while in 1886-87 only 305 out of every 1,000 matriculates were graduated.

The subjoined summary and tables will enable a further study of this subject to be made.

### SUMMARY OF MEDICAL INSTITUTIONS AND STUDENTS IN THE UNITED STATES AND CANADA, 1877—1887, INCLUSIVE.

| Institutions. | Regular. | Homœo. | Eclectic. | Ph. Med. | Miscell. | Fraud't. | Totals. |
|---|---|---|---|---|---|---|---|
| Total number of Medical Institutions embraced in this Report (*a*) | 174 | 25 | 34 | 8 | 4 | 13 | 258 |
| In the United States | 158 | 25 | 34 | 8 | 4 | 13 | 242 |
| In Canada | 16 | | | | | | 16 |
| Total number of Colleges now in existence (*b*) | 105 | 13 | 8 | 3 | | | 129 |
| In the United States | 93 | 13 | 8 | 3 | | | 117 |
| In Canada | 12 | | | | | | 12 |
| Total number of Institutions now extinct | 70 | 12 | 26 | 5 | 3 | 13 | 129 |
| In the United States | 66 | 12 | 26 | 5 | 3 | 13 | 125 |
| In Canada | 4 | | | | | | 4 |
| Total number of Colleges which now exact educational qualifications as a condition of matriculation (*c*) | 90 | 13 | 8 | 3 | | | 114 |
| Which formerly exacted such qualifications (*c*) | 41 | 4 | | | | | 45 |
| Total number of Colleges requiring attendance on three or more courses of lectures (*c*) | 37 | 5 | 1 | | | | 43 |
| Which formerly required such attendance (*c*) | 21 | 1 | | | | | 22 |
| Total number of Colleges which now recommend but do not exact three or more courses (*c*) | 45 | 7 | 4 | 1 | | | 57 |
| Which formerly made such recommendation (*c*) | 43 | 7 | 2 | 1 | | | 53 |
| Total number of Colleges which now have chairs of hygiene (*c*) | 91 | 13 | 8 | 2 | | | 114 |
| Which formerly taught this branch (*c*) | 32 | 7 | 3 | | | | 42 |
| Total number of Colleges which now have chairs of medical jurisprudence (*c*) | 88 | 13 | 8 | 3 | | | 112 |
| Which formerly taught this branch (*c*) | 49 | 8 | 4 | | | | 61 |
| Total number of Colleges which require a thesis | 30 | 3 | 4 | | | | 37 |
| Total number of Colleges for women only | 7 | 1 | | | | | 8 |
| In the United States | 5 | 1 | | | | | 6 |
| In Canada | 2 | | | | | | 2 |
| Total number of Colleges for both sexes | 20 | 8 | 9 | 2 | 1 | | 40 |
| Total number of Colleges for colored students only | 2 | | | | | | 2 |
| For both white and colored students | 1 | | | | | | 1 |

NOTES.—(*a*) "Total number of Institutions," includes seven (7) examining and licensing bodies which do not give instruction; and three (3) schools which do not confer degrees.

(*b*) Does not include those specified in note *a*.

(*c*) "Now" and "formerly" have reference respectively to the periods before and since the enforcement of the Schedule of Minimum Requirements, namely, the close of the sessions of 1882-1883.

### LIST OF COLLEGES IN THE UNITED STATES AND CANADA NOW IN EXISTENCE, BY STATES.

1. Medical College of Alabama, Mobile.
2. *Medical Department Arkansas Industrial University, Little Rock.
3. Cooper Medical College, San Francisco.
4. Medical Department, University of California, San Francisco.
5. California Medical College, San Francisco.
6. Hahnemann Medical College of San Francisco.
7. College of Medicine, University of Southern California, Los Angeles,
8. Toronto University, Medical Faculty, Toronto.
9. Royal College of Physicians and Surgeons, Kingston.
10. Medical Department, Weston University, London.
11. Woman's Medical College, Toronto.
12. Kingston Women's Medical College, Kingston.
13. McGill University, Faculty of Medicine, Montreal.
14. École de Medicine et de Chirurgie, Montreal.
15. Laval University, Medical Departments, Quebec and Montreal.

16. University of Bishop's College, Faculty of Medicine, Montreal.
17. Halifax Medical College, Halifax.
18. Dalhousie University, Faculty of Medicine, Halifax.
19. Manitoba Medical College, Winnipeg.
20. University of Denver, Medical Department, Denver.
21. Medical Department, University of Colorado, Boulder.
22. Gross Medical College, Denver.
23. Medical Department, Yale College, New Haven.
24. National Medical College, Washington.
25. University of Georgetown, Medical Department, Washington.
26. Howard University, Medical Department, Washington.
27. Medical Department, National University, Washington.
28. Medical College of Georgia, Augusta.
29. Atlanta Medical College, Atlanta.
30. Georgia College of Eclectic Medicine and Surgery, Atlanta.
31. Southern Medical College, Atlanta.
32. Rush Medical College, Chicago.
33. Chicago Medical College, Chicago.
34. Hahnemann Medical College and Hospital, Chicago
35. Bennett College of Eclectic Medicine and Surgery, Chicago.
36. Woman's Medical College of Chicago.
37. Chicago Homœpathic Medical College, Chicago.
38. College of Physicians and Surgeons, of Chicago.
39. Quincy College of Medicine, Quincy.
40. Physio-Medical Institute, Chicago.
41. Physio-Medical College of Indiana, Indianapolis.
42. Medical College of Indiana, Indianapolis.
43. Central College of Physicians and Surgeons, Indianapolis.
44. Fort Wayne College of Medicine, Fort Wayne.
45. Indiana Eclectic Medical College, Indianapolis.
46. Curtis Physio-Medical Institute, Marion.
47. College of Physicians and Surgeons, Keokuk.
48. Medical Department, State University of Iowa, Iowa City.
49. Homœpathic Medical Department, State University of Iowa, Iowa City.
50. Iowa College of Physicians and Surgeons, Des Moines.
51. University of Kansas, Medical Department, Lawrence.
52. University of Louisville, Medical Department, Louisville.
53. Kentucky School of Medicine, Louisville.
54. Louisville Medical College, Louisville.
55. Hospital College of Medicine, Louisville.
56. Medical Department, Tulane University of Louisiana, New Orleans.
57. Medical School of Maine, at Bowdoin College, Brunswick.
58. *Portland School for Medical Instruction, Portland.
59. University of Maryland, School of Medicine, Baltimore.
60. College of Physicians and Surgeons, Baltimore.
61. Baltimore Medical College, Baltimore.
62. Woman's Medical College, of Baltimore.
63. Baltimore University, School of Medicine, Baltimore.
64. Harvard University, Medical School, Boston.
65. Boston University, School of Medicine, Boston.
66. College of Physicians and Surgeons, Boston.
67. Department of Medicine and Surgery, University of Michigan, Ann Arbor.
68. Homœpathic Medical College, University of Michigan, Ann Arbor.
69. Detroit College of Medicine, Detroit.
70. Minnesota Hospital College, Minneapolis.
71. Minneapolis College of Physicians and Surgeons, Minneapolis.
72. St. Paul Medical College, St. Paul.
73. Minnesota Homœpathic Medical College, Minneapolis.
74. Missouri Medical College, St. Louis.
75. St. Louis Medical College, St. Louis.
76. Medical Department, University of the State of Missouri, No. 1, Columbia.
77. Homœpathic Medical College of Missouri, St. Louis.
78. Kansas City Medical College, Kansas City.
79. St. Louis College of Physicians and Surgeons, St. Louis.
80. American Medical College, St. Louis.
81. Northwestern Medical College, of St. Joseph.
82. University of Kansas City, Medical Department, Kansas City.
83. St. Joseph Medical College, St. Joseph.
84. Beaumont Hospital Medical College, St. Louis.
85. Omaha Medical College, Omaha.
86. Dartmouth Medical College, Hanover.
87. College of Physicians and Surgeons in the City of New York.
88. Albany Medical College, Albany.
89. University of the City of New York, Medical Department, New York.
90. Medical Department, University of Buffalo.
91. Long Island College Hospital, Brooklyn.
92. New York Homœpathic Medical College, New York.
93. Bellevue Hospital Medical College, New York.
94. New York Medical College and Hospital for Women, New York.

95. Eclectic Medical College of the City of New York.
96. Woman's Medical College of the New York Infirmary, New York.
97. College of Medicine of Syracuse University, Syracuse.
98. Medical Department of Niagara University, Buffalo.
99. Leonard Medical School, Raleigh, N. C.
100. Medical College of Ohio, Cincinnati.
101. Western Reserve University, Medical Department, Cleveland.
102. Eclectic Medical Institute, Cincinnati.
103. Starling Medical College, Columbus.
104. Homœpathic Hospital College, Cleveland.
105. Cincinnati College of Medicine and Surgery, Cincinnati.
106. Miami Medical College, Cincinnati.
107. Medical Department, University of Wooster, Cleveland.
108. Pulte Medical College, Cincinnati.
109. Columbus Medical College, Columbus.
110. American Eclectic Medical College, Cincinnati.
111. Toledo Medical College, Toledo.
112. Northwestern Ohio Medical College, Toledo.
113. Woman's Medical College, of Cincinnati.
114. Medical Department, Willamette University, Portland, Oregon.
115. Medical Department, Oregon State University, Portland.
116. University of Pennsylvania, Department of Medicine, Philadelphia.
117. Jefferson Medical College, Philadelphia.
118. Hahnemann Medical College and Hospital, Philadelphia.
119. Woman's Medical College of Pennsylvania, Philadelphia.
120. Medico-Chirurgical College, of Philadelphia.
121. Western Pennsylvania Medical College, Pittsburgh.
122. Medical College of the State of South Carolina, Charleston.
123. Medical Departments of the University of Nashville and Vanderbilt University, Nashville.
124. Medical Department, University of Tennessee, Nashville.
125. Meharry Medical Department of Central Tennessee College, Nashville.
126. Memphis Hospital and Medical College, Memphis.
127. Medical Department of the University of Vermont, Burlington.
128. University of Virginia, Medical Department, Charlottesville.
129. Medical College of Virginia, Richmond.

---

*Teaching Schools. Do not grant degrees.

LIST OF EXAMINING AND LICENSING BODIES.

1. Medical Association of the State of Alabama.
2. Illinois State Board of Health.
3. Iowa State Board of Medical Examiners.
4. Minnesota Medical Examining Board.
5. Mississippi StateeMedical Association, Board of Censors.
6. Board of Medical Examiners of North Carolina.
7. Medical Examining Board of Virginia.

---

The Board met pursuant to adjournment. Dr. W. A. Haskell was re-appointed by Governor Oglesby as a member of the Board, and as his own successor.

At the annual election of officers for the ensuing year, Dr. W. A. Haskell was re-elected President, and Dr. John H. Rauch was re-elected Secretary.

After the transaction of some further routine business, the Board adjourned.

